# Perceived stress is related to health-related quality of life in patients with chronic rhinosinuitis

**DOI:** 10.1186/2045-7022-3-S2-O13

**Published:** 2013-07-16

**Authors:** Ana Pangercic, Danijela Markota, Maja Grgec, Andro Kosec, Dejan Tomljenovic, Livije Kalogjera

**Affiliations:** 1 University Clinical Hospital "Sestre Milosrdnice", Zagreb, Croatia; 2 Zagreb School Of Medicine, Zagreb, Croatia; 3 University Clinical Hospital "Sestre Milosrdnice", ENT, Zagreb, Croatia

## Background

Chronic rhinosinusitis (CRS) is a common disorder with a significant impact on health-related quality of life (HRQL). Due to symptoms based diagnosis, disease severity is usually estimated using questionnaires which evaluate subjective scores on severity of symptoms and deterioration of HRQL, like Sino-Nasal Outcome Test-22 Questionnaire (SNOT-22). Objective severity staging is rather based on computerized tomography (CT) scores than on severity of inflammation. There is recent evidence that perceived stress has significant impact on asthma incidence and hospitalization, as well as on allergic rhinitis. As CRS is comorbidity of asthma and allergic rhinitis, we hypothesized that perceived stress may have impact on severity of CRS. The aim of the study is to correlate objective and subjective outcome measures with perceived stress.

## Method

The study was conducted in 29 patients with CRS, with and without nasal polyps, scheduled for surgical treatment. After giving their informed consent, patients filled in SNOT-22 and Measure of Psychological Stress for assessment of perceived stress. We divided SNOT-22 Questionnaire into questions related to nasal symptoms (SNOT-22 nasal) and to other symptoms (HRQL SNOT-22). These results were correlated with Lund-MacKay CT score and semi-quantitative scoring of inflammatory cells infiltration of sinus mucosa.

## Results

There are significant positive correlations between HRQL SNOT-22 set of questions and MPS score (Pearson test, r=0,49, p=0,008) and between total SNOT-22 and MPS score (r=0,45, p=0,013). Correlation between scores of nasal and HRQL SNOT-22 is also significant (r=0,53, p=0,003) There is also significant correlation between scores of eosinophilic and mononuclear of infiltration (r=0,63, p=0,001). The only difference between CRSwNP and CRSsNP patients is significantly higher mononuclear infiltration in CRSsNP, and higher CT score in CRSwNP.

## Conclusion

Results are suggesting that subjective scoring of disease severity is related to perceived stress in CRS patients. As no correlation was found between perceived stress and inflammation severity or CT severity scores, further research in order to evaluate possible cause-and-effect relationship should be undertaken. Our results suggest that CRS staging based on combined symptoms and HRQL scoring, may be moderated by patients' stress exposure and perception.

